# Sex Dimorphism in the Metabolome of Metabolic Syndrome in Morbidly Obese Individuals

**DOI:** 10.3390/metabo12050419

**Published:** 2022-05-06

**Authors:** Serena Pisoni, Vannina G. Marrachelli, Jose M. Morales, Sabrina Maestrini, Anna M. Di Blasio, Daniel Monleón

**Affiliations:** 1 Department of Pathology, Medicine and Odontology Faculty, University of Valencia, 46010 Valencia, Spain; serena.pisoni@uv.es (S.P.); j.manuel.morales@uv.es (J.M.M.); 2 Department of Physiology, Medicine and Odontology Faculty, University of Valencia, 46010 Valencia, Spain; vannina.gonzalez@uv.es; 3 Clinical Hospital Research Foundation-INCLIVA and CIBERFES, 46010 Valencia, Spain; 4 Laboratory of Molecular Genetics, Istituto Auxologico Italiano IRCCS, 20145 Milano, Italy; s.maestrini@auxologico.it (S.M.); a.diblasio@auxologico.it (A.M.D.B.)

**Keywords:** metabolic syndrome, severe obesity, metabolomics, sex dimorphism, age dependence

## Abstract

Adult morbid obesity is defined as abnormal or excessive fat accumulation, mostly resulting from a long-term unhealthy lifestyle. Between 10% and 30% of people with obesity exhibit low cardiometabolic risk. The metabolic syndrome has been suggested as an indicator of obesity-related metabolic dysregulation. Although the prevalence of obesity does not seem to be sex-related and metabolic syndrome occurs at all ages, in the last few years, sex-specific differences in the pathophysiology, diagnosis, and treatment of metabolic syndrome have received attention. The aim of this study was to determine the prevalence of metabolic syndrome and its components in different sex and age groups in people with metabolic unhealthy obesity and to compare them with people with metabolic healthy obesity. We analyzed the metabolome in 1350 well-phenotyped morbidly obese individuals and showed that there is a strong sex-dependent association of metabolic syndrome with circulating metabolites. Importantly, we demonstrated that metabolic dysregulation in women and men with severe obesity and metabolic syndrome is age-dependent. The metabolic profiles from our study showed age-dependent sex differences in the impact of MetS which are consistent with the cardiometabolic characterization. Although there is common ground for MetS in the metabolome of severe obesity, men older than 54 are affected in a more extensive and intensive manner. These findings strongly argue for more studies aimed at unraveling the mechanisms that underlie this sex-specific metabolic dysregulation in severe obesity. Moreover, these findings suggest that women and men might benefit from differential sex and age specific interventions to prevent the adverse cardiometabolic effects of severe obesity.

## 1. Introduction

Adult morbid obesity is defined as abnormal or excessive fat accumulation, mostly resulting from a long-term unhealthy lifestyle. Since 1975, worldwide obesity has nearly tripled and today the World health organization (WHO) considers it a global epidemic, representing a significant risk factor for the appearance of pathological states and mortality. It is importantly related to cardiovascular disease and diabetes, but also to cancer and chronic diseases, including osteoarthritis, liver and kidney disease, sleep apnea, and depression [1]. People who suffer from these pathological consequences of obesity are considered metabolically unhealthy obese (MUHO) and represent the most common condition. As a counterpart, a less diffuse phenotype of obesity (metabolically healthy obese, MHO) is characterized by a favorable inflammatory and metabolic profile [2]. MHOs represent between 10% and 30% of people with obesity and exhibit low cardiometabolic risk (higher insulin sensitivity, normal blood pressure, lower lipid levels of triglycerides (TGs), low-density lipoproteins, and higher levels of high-density lipoproteins). Although the clinical relevance of these subgroups remains a matter of debate [3], these discordant metabolic phenotypes represent a unique and understudied opportunity to examine the relationship between adipose tissue expansion and metabolic disease.

Currently, metabolic syndrome (MetS) is the most common health problem related to MUHO and the accumulation of abdominal fat. As stated in the National Cholesterol Education Program Adult Treatment Panel III (NCEP-ATP III) and the International Diabetes Federation [4,5], MetS is defined as the combination of three or more metabolic alterations such as abdominal obesity, hypertension, hyperglycemia/diabetes mellitus 2 (DM2), hypertriglyceridemia, and low high-density lipoprotein (HDL) cholesterol. MetS has dynamic aspects regarding, for example, the order in which its components occur and their exposure time, or the time that elapses between the first and the second event and if this time changes depending on the order of appearance of each component [6,7]. Moreover, the five components of MetS involve many metabolic pathways, many of them involved in the pathogenesis of the metabolic disease. Hence, the clinical consequences of MetS seem to reflect not the simple sum of each metabolic alteration, but their synergistic effect that aggravates the outcome [8], collectively increasing the risk of developing diabetes and cardiovascular diseases. The MetS has been suggested as an indicator of obesity-related metabolic dysregulation [8].

The prevalence of obesity does not seem to be sex-related and MetS occurs at all ages. Nevertheless, in the last few years, sex-specific differences in the pathophysiology, diagnosis, and treatment of MetS have received attention and have been attributed to variations in body fat distribution and endocrine profiles. Moreover, it has been reported that the prevalence of each MetS’ single component is sex [9,10] and age-dependent, representing an important health problem, especially in men and postmenopausal women. This is due, among others, to significant changes in the distribution of body adipose tissue with age. Males tend to accumulate fat more viscerally (especially abdominal). Females, despite having an overall and physiologically higher body fat content than men have a different distribution of adipose tissue. This tends to be present before menopause and accumulates mainly at the subcutaneous level. After menopause, it switches to a more abdominal accumulation with up to twice the amount of visceral adipose tissue than in premenopausal women [11]. Sex hormones appear to play a predominant role in these changes and in the biochemical differences between males and females. The physiological decline in estriol (a type of estrogen with cardio-protective activity) in menopause contributes directly to the onset of cardiovascular risk factors. This is because sex-hormone levels seem to be particularly associated with insulin amount, insulin sensitivity, and obesity, which in turn are strongly related to the MetS [12]. Accordingly, men and women of distinct ages may be characterized by different MetS combinations and variations in component clusters [13]. Despite all evidence in favor of sex, the studies of sex in the manifestation of MetS morbid obesity are scarce.

The metabolome, the set of metabolites in a biological sample, mirrors the products of the genome, transcriptome, and proteome and reflects any environmental or endogenous manifestations. Comprehensive metabolic profiling of human serum could provide an exhaustive view of the metabolic pathways affected by severe obesity and metabolic disease. Over the last decade, numerous reports and reviews have addressed the metabolic changes associated with obesity in both human and animal models (for a systematic review, see reference [14]). Sex hormones are a major factor in these differences but knowledge on sex-specific metabolic dysregulation in obesity is sparse and the studies focused on them are small-scaled or not focused on humans [14].

The aim of this study was to determine the prevalence and metabolomic profile of MetS among different sex and age subgroups in people with metabolic unhealthy obesity, and to compare them with people with metabolic healthy obesity.

## 2. Results

### 2.1. Population Characteristics and MetS Incidence

Our study population included 1350 individuals. All of them had extreme obesity: Body Mass Index (BMI) > 40 kg/m^2^, waist circumference ≥90 cm in women and ≥112 cm in men, and moderate to high Waist to Hip Ratio (WHR). The cohort was composed of 65.5% of women and 34.5% of men with ages between 19 and 85 years. The mean age was 53.7 years with a significant difference in mean age between males and females (Table 1). The anthropometric, fat mass and energy-expenditure parameters also showed statistically significant differences between men and women, as expected. Finally, clinical biochemistry demonstrates that, despite the extreme effect of severe obesity, cholesterol, triglycerides, and insulin metabolisms differ between men and women.

The anthropometric and clinical characterization of our cohort showed a prevalence of MetS of 80.5% which is expected in a severe obesity context (Table 2). The analysis of the different components of MetS was stratified by sex and age. This, along with the prevalence of Mets.5 and MHO demonstrated a higher prevalence of MetS after 54 years in both sexes. However, the increase in prevalence with age was much sharper in women, whereas it was almost neglectable in men. This increase in the prevalence of MetS after the age of 54 is parallel to changes in the anthropometric parameters such as waist circumference (WC), WHR, and BMI (Table S1, Supplementary Materials). MetS.5 was observed in 23.6% of the entire cohort with an increasing prevalence with age in both women and men. A percentage of 69.4% of women in the younger group (18–45) and 79.6% of women in the older group (55–85) displayed MetS, corresponding to an odds ratio of 1.72. Men belonging to the same ages displayed MetS with a prevalence of 79.6% and 89.7%, respectively, with an odds ratio of 2.22. We also analyzed the prevalence of MetS.5 to explore extreme effects and better differentiate between MHO and the disease of obesity in the context of extreme BMI. In men, the prevalence of MetS.5 was similar in both age groups with values around 25%, whereas in women, the older age group showed more than double the prevalence of the younger age group, rising from 11% to 23.2% (Table 2).

The analysis of individual metabolic components also showed differences strongly related to sex and age ranges in their contribution to the MetS pathological state. The percentage of individuals with hypertension and hyperglycemia/DM2 changed significantly between age groups. These differences also proved to be statistically relevant when comparing men and women. The percentage of individuals with Low-HDL was significantly different by age but its prevalence was comparable in both sexes. On the contrary, hypertriglyceridemia percentage did not show a relevant change between the two age ranges, but it did change notably with sex, indicating a strong influence of sex but not of age on the prevalence of this metabolic component (Table 2). Notably, there was an increasing trend in older women towards raised systolic and/or diastolic blood pressure compared with women in the younger age group. The percentage of men with hypertension was high and stable for both ages. Interestingly, the prevalence of a Low-HDL parameter decreased with age in both sexes.

### 2.2. The Metabolomic Differences between MHO and MetS Are Age-Dependent

**Figure 1:**
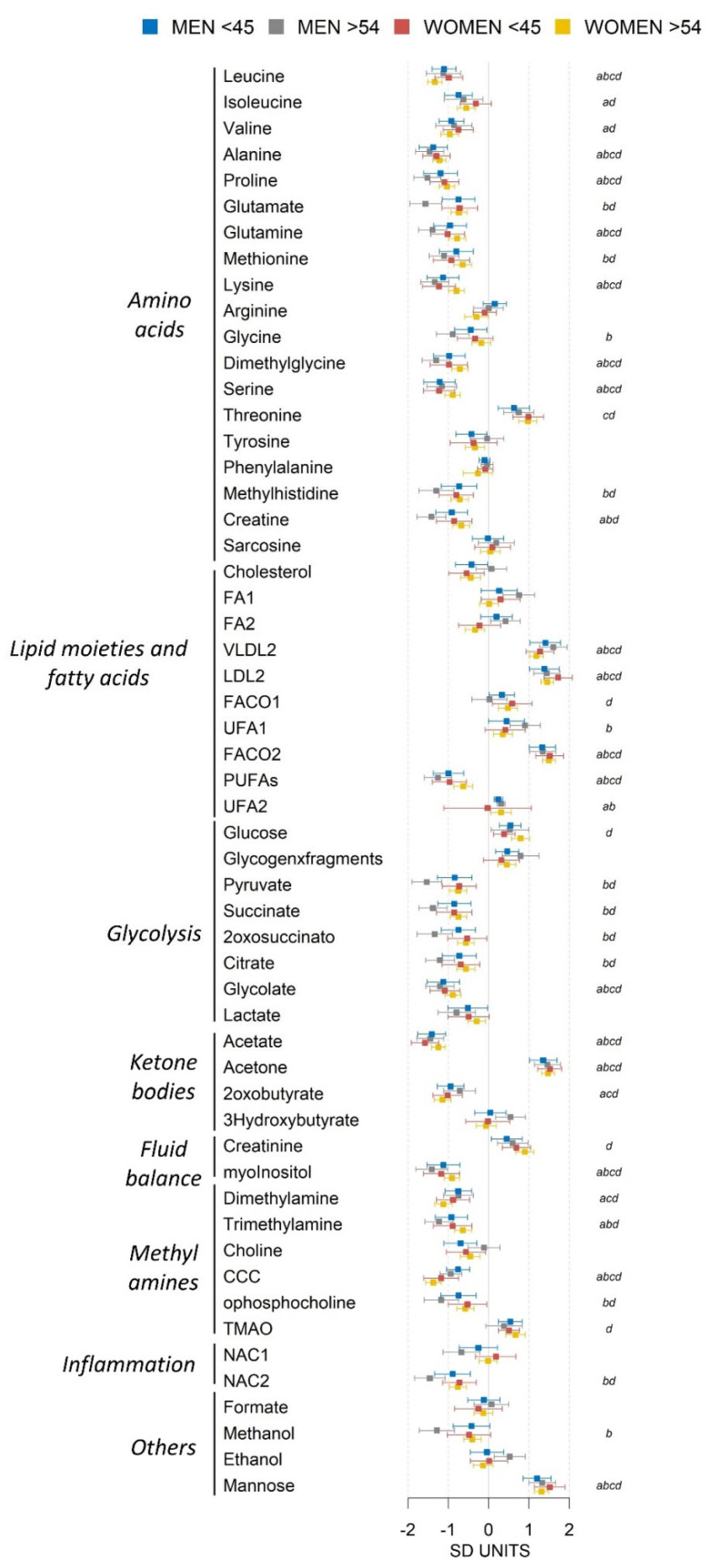
Mean differences and 90% confidence intervals between MetS and MHO expressed in SD units for men (age under 45 in blue, age over 54 in gray) and women (age under 45 in red, age over 54 in yellow) for all the metabolic features measured in the study. Multiple tests corrected statistical significance for the comparison between MHO and MetS (*p*-value below 0.00091) is marked with ‘*a*’ (men under 45 years), ‘*b*’ (men over 54 years), ‘*c*’ (women under 45 years) and ‘*d*’ (women over 54 years) in the last column. Key for NMR moieties: FA: fatty acids; FA1: CH3-; FA2: -CH2-; FACO1: -CH2CO; FACO2: -CH2CH2CO; UFA1: =CHCH2CH2-; UFA2 =CHCH2-; PUFAs: =CHCH2CH=; CCC: choline-containing compounds; TMAO: trimethylamine oxide; NAC1/2: acetyls in glycoproteins; LDL2: low-density lipoprotein 2; VLDL2: very-low-density lipoprotein 2.

Our metabolic profiling approach provided information on 55 well-defined spectral metabolic features (Figure S1, Supplementary Materials). All of them were assigned to unique metabolic components, although five of them, namely proline, glycolate, 2oxosuccinate, creatinine, and threonine were not confirmed by the ^1^H,^1^H-TOCSY spectra because of low signal-to-noise in the 2D spectra. We analyzed the metabolic differences associated with MetS in the different sex and age groups to identify sex-specific mechanistic clues and the age impact upon them. We evaluated the association of MetS with all 55 metabolic components. Figure 1 shows the mean difference and confidence intervals between MetS and MHO for the four different sex and age groups. Sixteen metabolic components showed statistically significant association with MetS in all four groups. In general, men in both age groups exhibited increasingly intense metabolomic differences between MHO and MetS. Women in the older group showed different, more intense changes that were closer to the men’s changes, compared to the younger group.

**Figure 2:**
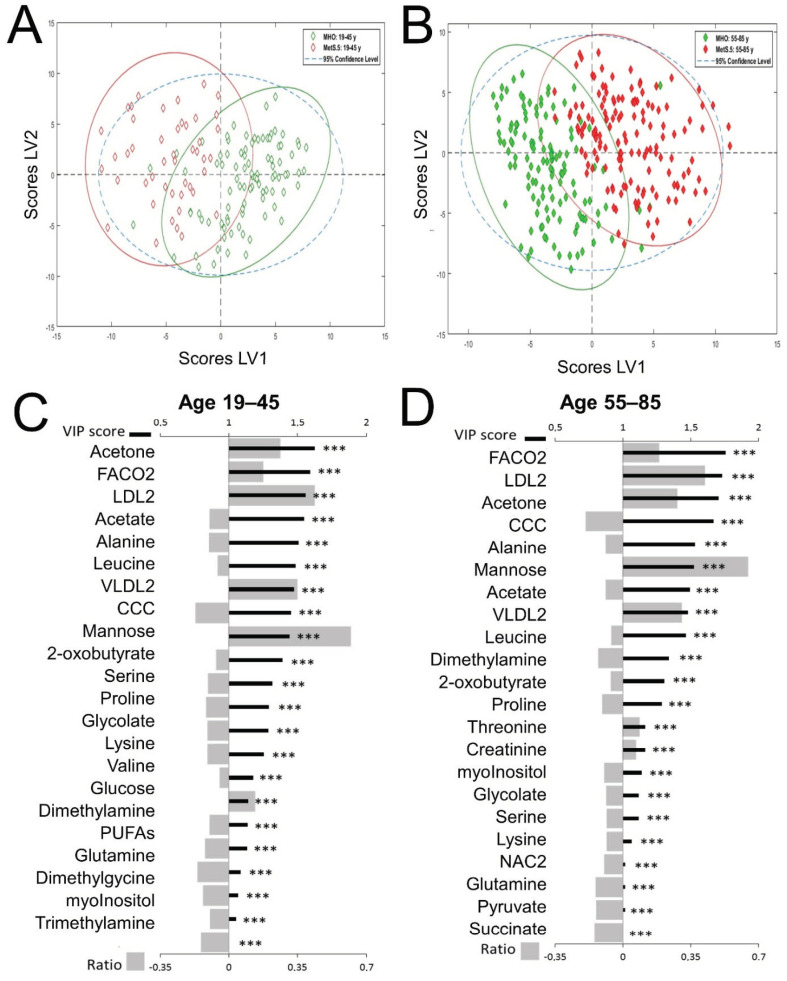
PLS-DA model for discrimination between MetS.5 and MHO by age range. All models were built using 2 latent variables (LV). All scores plots show MetS.5 samples in red and MHO samples in green with 95% confidence ellipse in the same colors. VIPs scores are represented as thick black lines (scale on the top) and relative fold change calculated as (Metabolite mean concentration of Mets.5—Metabolite mean concentration of MHO)/Metabolite mean concentration of MHO, are represented by grey bars (scale on the bottom). Significant alterations are indicated by *. Adjusted *p*-values (Student’s *t*-test corrected by Bonferroni method) to 0.000018 (***). (**A**). Scores plot for the age range 19 to 45 years model. Cross-validation parameters: RMSECV 0.304, R2CV: 0.582; ROC Curve AUC: 0.96. (**B**). Scores plot for the age range 55 to 85 years model. Cross-validation parameters: RMSECV 0.300, R2CV: 0.640; ROC Curve AUC: 0.97. (**C**). VIP score and relative fold change bar plot for the age range 19 to 45 years model. (**D**). VIP score and relative fold change bar plot for the age range 55 to 85 years model.

It is well known that MetS prevalence increases with age. To maximize the differences associated with age and to further select relevant metabolic components, we calculated PLS-DA models to discriminate between MHO and MetS.5 in the two age groups. VIP scores were used to identify the metabolic components contributing the most to the models. Finally, the normalized fold change between the mean for MetS.5 and the mean of MHO for each metabolic component with a VIP greater than 1 were calculated to identify the change sense and distinguish increases vs. decreases in variables. The PLS-DA analyses between MHO and MetS.5 conditions for both the age range 19–45 and the age range 55–85, as well as their comparison, revealed specific metabolic changes in MetS.5. The global metabolic profiles showed differences between MHO and MetS.5 at both age ranges (Figure 2A,B). Moreover, the combined criteria of VIP scores and relative fold change (Figure 2C,D) further refine the associations identified by the mean differences (Figure 1), and revealed common trends regardless of age in the impact of MetS.5, including carbonyls in fatty acids (FACO2), LDL particles, and acetate and derivatives. Although most of the metabolites were selected as relevant for MetS.5 in both age groups, the VIP revealed different contributions to the models. Acetone and acetate, both dicarboxylic molecules, were the metabolites most significantly associated with MetS.5 in younger ages in the entire population (Figure 2C), whereas in older ages FACO2 showed the most important contribution to the model (Figure 2D). Interestingly, polyunsaturated fatty acids (PUFAs) only contributed significantly to the model in the younger age group, whereas pyruvate and succinate, involved in the Krebs cycle, were only selected in the older age group.

### 2.3. Sex Influence on MetS Metabolomic Impact in the Context of Severe Obesity

**Figure 3:**
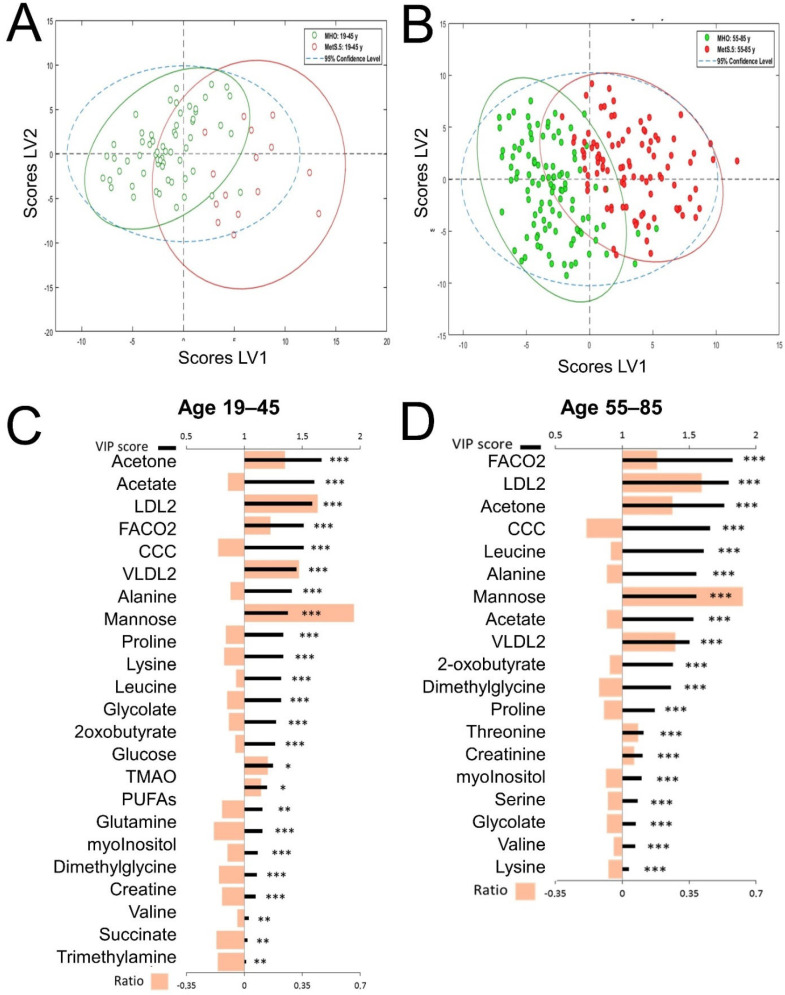
PLS-DA model for discrimination between MetS.5 and MHO by age range for women. All models were built using 2 latent variables (LV). All scores plots show MetS.5 samples in red and MHO samples in green with 95% confidence ellipse in the same colors. VIPs scores are represented as thick black lines (scale on the top) and relative fold change calculated as (Metabolite mean concentration of Mets.5—Metabolite mean concentration of MHO)/Metabolite mean concentration of MHO, are represented by grey bars (scale on the bottom). Significant alterations are indicated by *. Adjusted *p*-values (Student’s *t*-test corrected by Bonferroni method) to 0.00091 (*), 0.00018 (**), 0.000018 (***). (**A**). Scores plot for the age range 19 to 45 years model. Cross-validation parameters: RMSECV 0.273, R2CV: 0.534; ROC Curve AUC: 0.97. (**B**). Scores plot for the age range 55 to 85 years model. Cross-validation parameters: RMSECV 0.298 R2CV: 0.642; ROC Curve AUC: 0.98. (**C**). VIP score and relative fold change bar plot for the age range 19 to 45 years model. (**D**). VIP score and relative fold change bar plot for the age range 55 to 85 years model.

**Figure 4:**
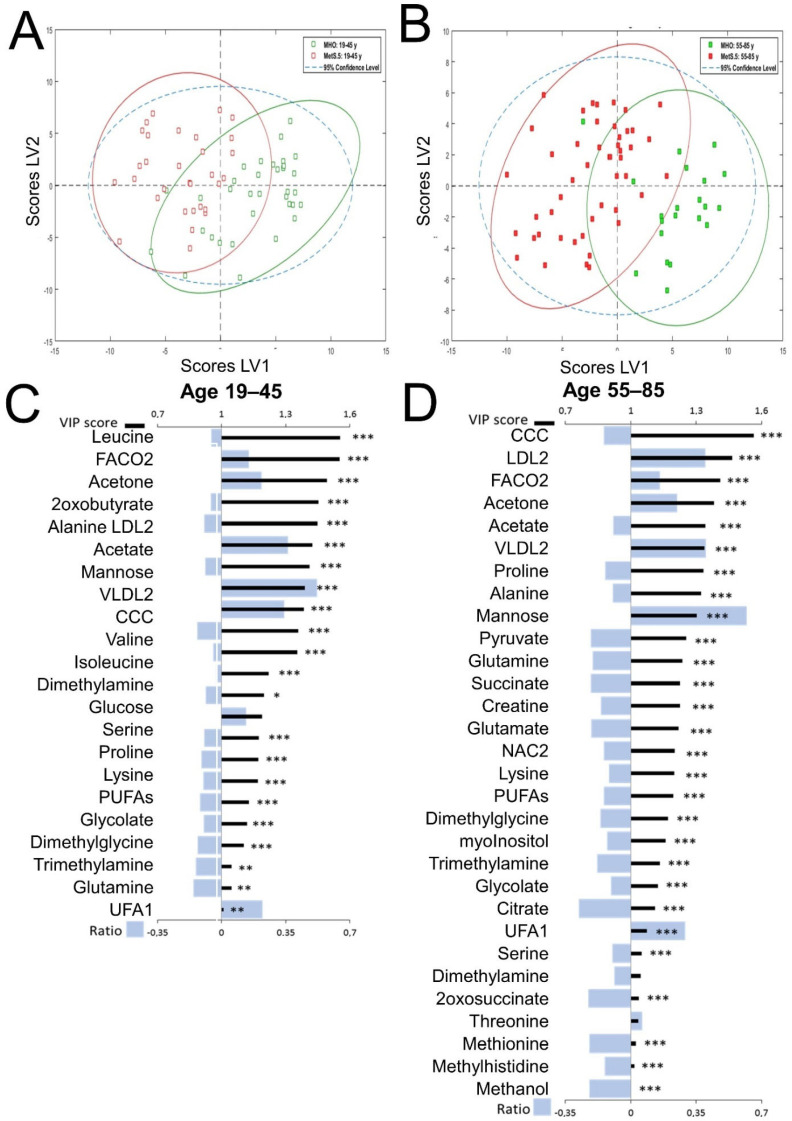
PLS-DA model for discrimination between MetS.5 and MHO by age range in men. All models were built using 2 latent variables (LV). All scores plots show MetS.5 samples in red and MHO samples in green with 95% confidence ellipse in the same colors. VIPs scores are represented as black thick lines (scale on the top) and relative fold change calculated as (Metabolite mean concentration of Mets.5—Metabolite mean concentration of MHO)/Metabolite mean concentration of MHO, are represented by grey bars (scale on the bottom). Significant alterations are indicated by *. Adjusted *p*-values (Student’s *t*-test corrected by Bonferroni method) to 0.00091 (*), 0.00018 (**), 0.000018 (***). (**A**). Scores plot for the age range 19 to 45 years model. Cross-validation parameters: RMSECV 0.341, R2CV: 0.534; ROC Curve AUC: 0.95. (**B**). Scores plot for the age range 55 to 85 years model. Cross-validation parameters: RMSECV 0.304, R2CV: 0.566; ROC Curve AUC: 0.95. (**C**). VIP score and relative fold change bar plot for the age range 19 to 45 years model. (**D**). VIP score and relative fold change bar plot for the age range 55 to 85 years model.

Since most of the clinical parameters of MetS showed statistically significant differences between men and women in both age groups, we applied the same strategy for analyzing the metabolomic profiles in the four sex and age subgroups (Figure 3 and Figure 4). The comparison of the global metabolomes at younger and older ages for women (Figure 3) and men (Figure 4) also showed discrimination and global differences (scores plots in Figure 3A,B for women and Figure 4A,B for men). The MetS.5 metabolomic impact at younger ages was different between women (Figure 3C) and men (Figure 4C). Although when comparing age groups regardless of sex (Figure 2) the number of metabolites with a VIP greater than one were comparable, the same number differed in the sex-stratified analysis. Women showed fewer metabolites with a VIP greater than one in the older group and men show the opposite trend. Although in all the comparisons carbonyls in fatty acids (FACO2) were among the top four VIP scores, the change in this metabolic component contribution with age was much sharper in women (from fourth position to first position) than in men (from second position to third position). Host-microbiota co-metabolites also showed different trends between men and women with choline compounds as the top contributor in the model for older men but as a medium contributor (fourth and fifth position for younger and older ages respectively) in the women models. Although changes in the contributions to the models showed interesting age-related trends and MetS.5 differences between men and women, most of the relative fold changes of each metabolite remained similar in all the MetS.5 analyses for the different groups, indicating a common metabolic impact of MetS. Among them, mannose showed the highest fold change and was statistically significant in all the comparisons. Other metabolites that also showed a VIP greater than one and statistically significant differences between MHO and MetS.5 in severe obesity included FACO2, acetone, alanine, LDL2, VLDL2, acetate, mannose, choline-containing compounds (CCC), serine, proline, lysine, and glycolate.

### 2.4. MetS Produces Metabolomic Changes in Male Older Than 55 That Are Different from Those in Other Subgroups

**Figure 5:**
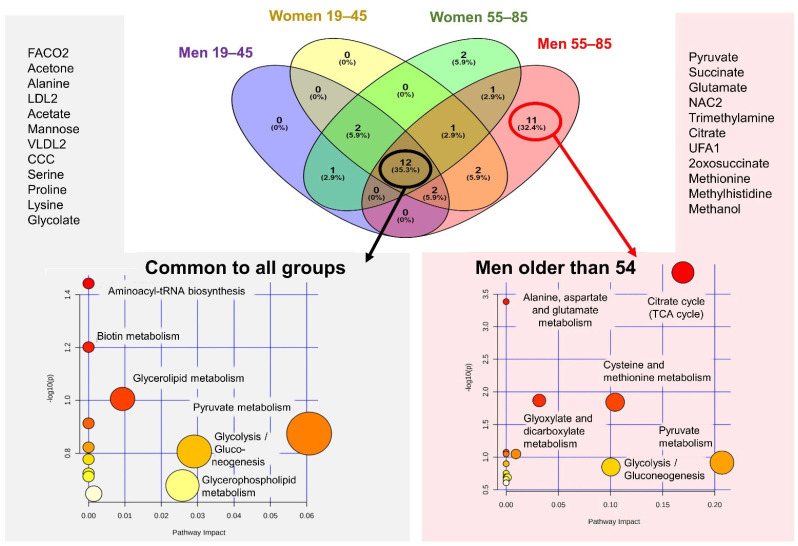
Metabolic pathways affected in MetS5 in all groups. Venn diagram of the metabolites identified as altered in MetS.5 with respect to MHO in the different age and sex groups, and pathway enrichment analysis of all groups’ intersections and the metabolic specific for men older than 54. For each subgroup (represented by ovals of different colors) is shown the number of the metabolites that are common to two, three or all the four groups (where ovals overlap) or those that are exclusive to each group (outer ovals). Metabolic pathway enrichment analysis for metabolites common to all groups (left, grey background) and exclusive for men over 54 years old (right, pink background) are also represented. Metabolic pathways whose name is indicated are significant (*p*-value lower than 0.05 after the adjustment using Holm-Bonferroni method and False Discovery Rate) and have a pathway impact value, calculated from pathway topology analysis, over 0. The x-axis indicates the impact on the route, while the y-axis indicates significant changes to a route. The pathways are represented as circles. The circle color indicates the significance level, from highest (red) to lowest (white) in the enrichment analysis. The circle size is proportional to the impact value of each road from the topology analysis.

The comparison of the specific profiles of MetS.5 in each age and sex subgroup by the Venn diagram (Figure 5) revealed minor differences with respect to age and sex, with variations in only up to two metabolites, except for men over 54 years where the variations included more metabolites. Twelve metabolites (35.3% of the total) resulted in common among all the subgroups and eleven metabolites (32.4% of the total) appeared specific for the subgroup of men over 54 years old (Figure 5). The impact of MetS.5 in this subgroup specifically affected up to 11 metabolites, including pyruvate, succinate, glutamate, acetyls in glycoproteins (NAC2), trimethylamine, citrate, unsaturated fatty acids (UFA1), 2-oxosuccinate, methylhistidine, and methanol. To gain insights into the metabolic mechanism of MetS in the context of severe obesity and the specific pathways affected differentially in older men, these selected metabolites were analyzed using Pathways enrichment analysis (MetaboAnalyst [15], Figure 5). Five metabolic pathways were statistically significant for the MetS.5 metabolites common to all group comparisons, including Pyruvate metabolism; Glycolysis/Gluconeogenesis; Glycerophospholipid metabolism; Fatty acid degradation; and Glycerolipid metabolism. The pathway enrichment analysis of the 11 metabolites (exclusive of the comparisons in men older than 54) included three additional pathways, namely, Citrate cycle (TCA cycle), Cysteine and methionine metabolism, and Glyoxylate and dicarboxylate metabolism, and it further confirmed the pathways identified in the common metabolomic profile.

## 3. Discussion

In this study, we examined the metabolomic impact of MetS in a well-phenotyped morbidly obese cohort and explored the sex dimorphism of this impact in two age groups, equivalent to pre- and post-menopause in women. We analyzed the differences in metabolome between MHO individuals and individuals who met all five criteria for MetS as extreme cases of MetS. These anthropometric, cardiometabolic, and metabolomic profiles of MetS in morbid obesity showed highly consistent sex and age differential patterns of effect. Our analysis allowed the identification of circulating traits that may underly the higher cardiometabolic disease rates commonly seen among older men. Our data demonstrate that, although morbid obesity represents an extreme case of adipose tissue growth, the metabolic impact is still modulated by sex and age. Consequently, the risk assessment in MetS patients with morbid obesity needs to be accordingly stratified. Causal analyses of these traits in relation to clinical endpoints are needed to understand whether they differentially affect cardiometabolic risk among obese males and females.

Overall, MetS prevalence in morbid obesity increased after the age of 54 in both sexes. However, the percentage of women with the five MetS criteria sharply increased after this age, compared to the modest increase observed in the same percentage of men. Interestingly, when the criteria were analyzed one by one, we observed parallel increases with age for both sexes in all the criteria except for hyperglycemia. Morbidly obese men exhibited higher rates of hyperglycemia than women of equivalent age with a sharp increase after the age of 54. In addition to a myriad of alterations, age-related declines in the function of sex hormones lead to alterations in adipocytes structure and quantity, fat tissue metabolism, and insulin sensitivity [16], which may be directly related to the doubling of the prevalence of MetS.5 in postmenopausal women, reaching that of men, where it remains relatively constant. Although some of these differences between women and men with MetS have been reported previously [17], this is the first time that they have been observed in the context of morbid obesity.

Metabolites are the endpoint of many biological and physiological processes and metabolomics may thereby provide snapshots of different physiological states. When measuring the differences between patients and controls, metabolomics allows for the detection of metabolic cores and pathways affected by the disease. By comparing these differences in different groups of sex and age, we can better understand how the disease develops in men and women. In this study, we observed variations in the way MetS impacts the metabolome of morbidly obese women and men. We analyzed the mean differences, multivariate PCA and PLS-DA models, VIP scores, and relative fold changes between metabolically healthy individuals and individuals with the five criteria for MetS. Most of the metabolites with a significant association with MetS and with a high contribution to the multivariate models remained the same for all the comparisons, suggesting a common ground for the development of extreme MetS regardless of age and sex. However, we observed larger differences between MetS and controls in men in both age groups and in older individuals of both sexes. MetS seemed to mostly affect the levels of mannose, carbonyls in fatty acids, acetone, LDL, VLDL, and acetate regardless of sex and age. These metabolites showed as significant or high VIP in the models in all the comparisons and represent a common signature of MetS in morbid obesity. We observed that high levels of mannose, the metabolite whose concentration shows the most relevant change in the pathological condition in all the Piancavallo cohort analyzed subgroups, were positively associated with insulin resistance and diabetes and were considerably implicated in the syndrome and its clinical complications [18]. As expected, lipid metabolism predominates in this common ground of MetS in morbid obesity. Other studies have reported associations of MetS with levels of branched-chain amino acids to be the most biologically relevant [19]. In the context of morbid obesity, these amino acids also show a strong statistical association with MetS but seem to be less significant than the lipid markers. Interestingly, the lipid profiles of women and men with MetS showed similar elements but with different importance. Metabolites with a potential bacterial origin are also among the top contributors to models and the most significantly associated with MetS. The gut microbiome has been reported as being able to influence susceptibility to obesity by altering the efficiency of energy harvest from the diet [20]. On the other hand, the gut bacterial ecosystem and the corresponding bacterial co-metabolism are altered by obesity and can promote low-grade inflammation and metabolic disease [20]. Some studies suggest that metabolites in the pathways from choline to trimethylamines are indicative of the gut microbiota status [21]. Our analysis showed that the levels of choline-containing compounds and methylamines were significantly decreased in morbidly obese individuals with MetS.5, compared to MHO. The pathway enrichment analysis of our data shows that the common ground of MetS in severe obesity affects fatty acid degradation, pyruvate metabolism, glycolysis and gluconeogenesis, and glycerophospholipid synthesis. Overall, our findings are in line with previous studies although we identified interesting new sex-related trends.

Different studies have characterized the major components of the MetS in women and men in different populations. Abdominal fat tissue produces free fatty acids and cytokines which in turn lead to insulin resistance, dyslipidemia, and high blood pressure. However, women seem to need a higher degree of adiposity to achieve the same metabolic disturbances because they exhibit a more favorable fat distribution. This fat distribution, probably regulated by estrogens, seems to confer protection beyond menopause [22]. However, it is unclear if this fat distribution effect is sustained in severe obesity. To better understand the role of estrogens in the metabolic impact of MetS in severe obesity, we analyzed separately women and men in group ages related to menopause transition. Menopause does not represent a clear-cut event in terms of global metabolism and physiology, and changes associated to menopause can linger for several years after the last ovulation. Menopause status should be examined using rigorous criteria which are not easily applicable in some patients’ groups [23]. For that reason, we defined two age groups, excluding the grey zone of ages between 45 and 54, to include a vast majority of pre-menopausal women and a vast majority of post-menopausal women. We observed a sharp increase in the prevalence of MetS in the post-menopause group, probably due to the associated body composition and structure changes and the decrease in protective effects from estrogens. Parallel to this increased incidence of MetS, the changes in metabolites associated with MetS were also of greater intensity. We observed that MetS affects acetate and acetone in premenopausal women, but not in postmenopausal women, suggesting that changes in fatty acid metabolism associated with menopause or MetS also affect ketone body production. It is well-known that fatty acids in the blood are converted to ketone bodies when the fatty acid concentration is high and the insulin is low [24]. Nevertheless, the excessive production of ketone bodies, as appears in diabetes, leads to their accumulation in the circulation and the development of ketosis and the acidosis of ketoacidosis. On the other hand, women in the post-menopausal age range with MetS exhibited higher levels of low-density lipoprotein cholesterol and carbonyls in fatty acids, further supporting alterations in lipid metabolism and suggesting the potential role of oxidative stress. Estrogens have antioxidant properties which protect mitochondrial integrity and function [25]. The metabolomic profile of obese women in an age group probably postmenopausal appears to represent a sort of intermediate situation between younger and older obese men. This suggests the protection against MetS that estrogens provide and that extends after menopause is also paralleled by less severe changes in the metabolome.

Our study confirms that the prevalence of MetS is higher in men than in women regardless of age and menopause. The impact on the different components of MetS was different between men and women in an age-dependent manner. At younger ages, men with MetS exhibited similar but more intense metabolomic changes than those observed in women in both age groups, which can probably be related to the different behavior in the individual MetS components. For example, there was an increase in the percentage of men who suffered from hyperglycemia/DM2 between younger and older groups. Our pathway enrichment analysis confirmed that in this subgroup the TCA cycle seems to be specifically affected and appears as the most significant pathway to the detriment of other relevant pathways in the context of severe obesity, such as fatty acid metabolisms or glycerophospholipids metabolism. The TCA cycle is a series of biochemical reactions that are utilized by all aerobic organisms to produce energy and is strongly related to mitochondrial function. We also observed in the discrimination models that in this older-men subgroup, the comparison between MHO and MetS.5 revealed choline-containing compounds as the greatest contributor to the models. These compounds have been suggested as proatherogenic by the inhibition of reverse cholesterol transport, which is also supported by the cholesterol profiles [21,26]. Finally, branched-chain amino acids, metabolites associated with metabolic disease in many studies, are only among the most contributing metabolites in the model for younger men, which suggests that metabolic disease in severe obesity more dramatically affects other pathways and the metabolic core.

There are several limitations to the current study. First, this was an observational study and our data do not provide information about causality mechanisms. Second, all individuals were of Western European descent and it is difficult to extrapolate these data to other populations. Third, we used metabolic syndrome as an indicator of cardiometabolic dysregulation. It is important to realize that there are various definitions for metabolic syndrome and this syndrome is heterogeneous. To optimize external validation, we used the most frequently used definition of the National Cholesterol Education Program ATP III. Our cohort included an unequal number of males and females. However, we stratified the analysis by sex and age to overcome potential bias. We did not evaluate actual menopause but group ages related to menopause transition, which may add some bias of the results in terms of interpretation. Numerous potential lifestyle-related factors could still influence the sex differences detected in this study beyond biological mechanisms, such as adverse dietary patterns, smoking behavior, or alcohol consumption.

In summary, we analyzed the metabolome in well-phenotyped morbidly obese individuals and showed that there is a strong sex-dependent association between MetS and circulating metabolites. Importantly, we demonstrated that metabolic dysregulation in women and men with severe obesity and MetS is age-dependent. The metabolic profiles from our study showed age-dependent sex differences in the impact of MetS which are consistent with the cardiometabolic characterization. Although there is a common ground for MetS in the metabolome of severe obesity, men older than 54 were affected in a more extensive and intensive manner. These findings strongly argue for more studies aimed at unravelling the mechanisms that underlie this sex-specific metabolic dysregulation in severe obesity. Moreover, these findings suggest that women and men might benefit from differential sex and age-specific interventions to prevent the adverse cardiometabolic effects of severe obesity.

## 4. Materials and Methods

### 4.1. Subjects and Study Design

This was a case-control study conducted on 1350 obese patients at the Division of General Medicine of the San Giuseppe Hospital, Istituto Auxologico Italiano (Piancavallo, Italy), recruited for diagnostic or therapeutic problems related to obesity or its morbidity during the period 2009–2010. All the voluntary participants were given an informed consent document with all the information concerning the study prior to taking part. In the session on 10 December 2008, the Ethics Review Committee of Istituto Auxologico Italiano (Milano), approved the studies, and all the participants gave written agreement to participate. Participants gave informed consent for their blood samples to be used for research studies. The individuals were extensively characterized by anthropometric measurements, diet evaluation, and complete metabolic characterization [27]. The anthropometric variables measured included body weight, height, and waist and hip circumference. Body composition, in terms of the percentage of fat body mass and fat-free body mass was determined using a bioelectrical impedance analysis (BIA101/S model; Akern, Florence, Italy). Patients with fluid overload, according to vectorial analysis were excluded to minimize errors in estimating fat body mass and fat-free body mass in severe obesity. Average daily caloric intake was evaluated by a 7-day recall standardized technique, details of which have been published previously [28]. The interviews were always carried out by two different trained dieticians who used a food frequency questionnaire and an atlas for the assessment of food-portion size. Moreover, all obese patients showing a negative difference between reported food intake and resting energy expenditure (REE) multiplied by 1.2 (as a cautious estimate of total energy expenditure) were considered as underreporting and thus, their case history was classified as unreliable. Diet and weight histories were assessed by interview according to standardized methodologies. REE (kcal/24 h) was determined following an overnight fast and at rest in a thermoregulated room (22–24 °C) using computed open-circuit indirect calorimetry (Sensormedics, Milan, Italy). Resting oxygen uptake and resting carbon dioxide production were measured by a ventilated canopy at 1-min intervals for 30 min and expressed as a 24-h value. According to The National Cholesterol Education Program Adult Treatment Panel III (NCEP-ATP III) and the International Diabetes Federation, the cohort was divided into people with MHO as controls, and people with MUHO as cases affected by MetS. The MUHO group was divided into subgroups based on the numbers of MetS parameters, from 3 to 5 (MetS.3, MetS.4, MetS.5), where MetS.5 represented the worst-case scenario of the disease. The study was focused on the comparison between this case subgroup and MHO. According to the literature, we divided both men and women into two big subgroups: fertile age, from 19 to 45 years, and postmenopausal age, from 55 to 85 years, for the metabolomics study of the MetS.5 profile related to sex and age.

### 4.2. Sample Collection and Preparation

Blood samples were obtained in the early morning fasting period eight to twelve hours prior to drawing the blood sample. Whole blood was collected in a covered test tube and was left undisturbed at room temperature from 30 min to 1 h to allow the blood to clot. The serum was then separated from the clot by centrifuging at 1000–2000× *g* for 10 min and immediately transferred with a pipette to a sterile 1.5 mL Eppendorf tube and frozen immediately at −80 °C until the NMR measurements were made.

### 4.3. Metabolite Quantification

A volume of 470 μL of serum sample was mixed with 30 μL of deuterium oxide (D_2_O) with internal reference to trimethylsilyl propionate (TSP) 2.5 mM and placed in a 5mm high-resolution NMR tube for SampleJet. The experiments were recorded at 310 K. The measurements of the samples from the different groups were carried out in a random order. Spectra were acquired using a Bruker Avance III DRX 600 spectrometer (Bruker GmbH, Rheinstetten, Germany) operating at a 1H frequency of 600.13 MHz (University of Valencia, Spain). For each serum sample, NMR spectra were acquired using a standard one-dimensional pulse sequence 1H ZGPR with water pre-saturation. The spectra were acquired using a 3.95 s acquisition time, 32 transients, a 14 ppm (8417 Hz) spectral width, and a relaxation delay of 2 s. The total acquisition time was 4 min 47 sec per sample. All spectra were processed using a MestReNova 14.1.1 (Mestrelab Research S.L., Santiago de Compostela, Spain). Each spectrum was Fourier-transformed, phased and baseline corrected. The spectra were referenced using the chemical shift of TSP at 0 ppm and were normalized to the aliphatic area (0.5–4.5 ppm) of the spectra. The data were imported into MATLAB R2019b (The MathWorks Inc., Natick, MA, USA) for additional processing and further analysis. Metabolite spin systems and resonances were identified by literature data, 1H,1H-TOCSY NMR spectra, and Chenomx resonances database (Chenomx NRM Suite 8.1, Chenomx Inc., Edmonton, AB, Canada). In addition, specialized NMR databases such as the Human metabolome database (HMDB) [29] were consulted together with the currently available literature on NMR-based metabolomics. NMR peaks were integrated and quantified using semi-automated in-house MATLAB peak integration routines. The final metabolite relative spectral abundance was calculated in arbitrary units as the peak area normalized to the total aliphatic spectral area, lipid excluded, to eliminate any differences in the metabolite total concentration.

### 4.4. Statistical Analysis

Chemometrics statistical analyses were developed using in-house MATLAB scripts and the PLS Toolbox 6.7 (Eigenvector Research, Inc., Wenatchee, WA, USA). Metabolite levels were computed from the raw (untransformed) data and normalized to the standard deviation in all the samples for obtaining z-scores. Principal component analysis (PCA) and projection to latent structures for discriminant analysis (PLS-DA) were applied to the metabolomic vectors of each sample. From the results of PLS-DA data analysis, Variable Importance in Projection (VIP) scores and respective relative fold changes between MetS.5 and MHO were calculated for each component (metabolite). tStudent’s test was used to determine the statistical significance of the differences between the means of the case and the control in both the women and men groups, and ANOVA was used to estimate the differences between the age categories. Results were cross-validated by performing the Venetian blind technical replications process 10 times to evaluate the accuracy of each classification model. The results of the cross-validation were evaluated by the RMSECV, R2 and the area under the receiver–operator curve (ROC) for cross-validation. Permutation tests were used for evaluating the significance of the models. All calculated models were significant at the 95% confidence level. A post hoc analysis based on the Bonferroni multiple comparison test was performed and a chi-squared test was used for comparative proportions. The significance level was *p* < 0.05 (+), 0.01 (++), 0.001 (+++). Corresponding adjusted *p*-values (Student’s *t*-test corrected by Bonferroni method) were 0.00091 (*), 0.00018 (**), 0.000018 (***). The statistical associations were adjusted for relevant variables and potential covariates using software SPSS, Matlab, R, and MetaboAnalyst. The categorical variables were analyzed by percentages.

## Figures and Tables

**Table 1:** Anthropometric characteristics of Piancavallo cohort data in total and separated by sex.

Variables	All Cohort (*n* = 1350)	Males (*n* = 465)	Females (*n* = 885)	*p*-Value
Age (years)	53.66 ± 13.84	50.34 ± 13.67	55.41 ± 13.61	+++
Height (cm)	162.55 ± 10.5	172.96 ± 7.75	157.08 ± 7.06	+++
Weight (kg)	126.54 ± 23.85	142.89 ± 24.07	117.96 ± 18.7	+++
BMI (kg m^−2^)	47.7 ± 6.54	47.63 ± 6.56	47.74 ± 6.52	
Waist circumference (cm)	133.27 ± 14.61	142.8 ± 13.1	128.25 ± 12.75	+++
Hip circumference (cm)	140.68 ± 14.45	139.34 ± 15.47	141.39 ± 13.82	
WHR (cm/cm)	0.95 ± 0.1	1.03 ± 0.07	0.91 ± 0.08	+++
Systolic blood pressure (mm Hg)	138.68 ± 17.61	140.9 ± 17.46	137.51 ± 17.58	
Diastolic blood pressure (mm Hg)	82.56 ± 9.31	84.42 ± 9.07	81.58 ± 9.28	+++
SatO2	93.51 ± 2.83	93.24 ± 2.47	93.65 ± 3	
HOMA-IR	4.39 ± 3.27	4.88 ± 2.85	4.14 ± 3.43	
s-Glucose (mg/dL)	112.29 ± 35.73	112.89 ± 34.05	111.98 ± 36.57	
s-LDL Cholesterol (mg/dL)	120.19 ± 34.87	119.53 ± 35.93	120.53 ± 34.29	
s-HDL Cholesterol (mg/dL)	42.67 ± 12.4	36.86 ± 9.68	45.73 ± 12.58	+++
s-Triglycerides (mg/dL)	144.1 ± 62.13	157.05 ± 60.92	137.3 ± 61.68	+++
s-Insuline (mU/L)	15.8 ± 9.4	17.95 ± 8.96	14.59 ± 9.42	+++
cc_H_2_O tot	39.14 ± 15.75	43.59 ± 4.38	36.81 ± 18.76	+++
cc_fat mass	49.52 ± 19.84	41.52 ± 5.7	53.72 ± 23.05	+++
cc_free-fat mass	51.61 ± 18.1	58.21 ± 6	48.16 ± 21.11	+++
cc_muscular mass	29.64 ± 11.51	33.56 ± 6.49	27.59 ± 12.9	+++
Basal metabolic rate	1995.77 ± 460.24	2376.72 ± 439.62	1777.38 ± 302.61	+++
Resting energy expenditure X day	2074.92 ± 457.13	2540.59 ± 395.5	1807.6 ± 207.8	+++
Resting energy expenditure%	96.55 ± 11.14	93.74 ± 10.69	98.16 ± 11.08	+++

Comparison and analysis to evaluate MetS in people who suffer from morbid obesity. Numbers data are reported as mean ± SD (Standard deviation). *p*-values are given for the difference in values between the sexes: *p* < 0.001 (+++). Abbreviations: BMI, body mass index; WHR, waist-to-hip ratio; SatO2, oxygen saturation; HOMA-IR, homeostasis model of assessment for insulin resistance; cc_, calorimetric calculation; s-, serum.

**Table 2:** Piancavallo cohort composition and cardiometabolic characterization.

Piancavallo Cohort Composition *n* (%)		Total			*p*-Value Age Ranges	Women			Men			*p*-Value Women vs. Men (by Age Ranges)
		All Ages 1350	Range 19–45 363 (26.9%)	Range 55–85 723 (53.6%)		All Ages 885 (65.5%)	Range 19–45 196 (22.1%)	Range 55–85 520 (58.8%)	All Ages 465 (34.5%)	Range 19–45 167 (35.9%)	Range 55–85 203 (43.7%)	
Metabolic profile	MHO	264 (19.5%)	94 (25.9%)	127 (17.6%)		196 (22.1%)	60 (30.6%)	106 (20.4%)	68 (14.6%)	34 (20.6%)	21 (10.3%)	
	MetS	1086 (80.5%)	269 (74.1%)	596 (82.4%)		689 (77.8%)	136 (69.4%)	414 (79.6%)	397 (85.4%)	133 (79.6%)	182 (89.7%)	
	MetS.5	256 (23.6%)	46 (17.1%)	144 (24.2%)		153 (22.2%)	15 (11%)	96 (23.2%)	103 (25.9%)	31 (23.3%)	48 (26.4%)	
Metabolic components	Hypertension	1100 (81.5%)	267 (73.6%)	611 (84.5%)	5.8 × 10^−7+++^	698 (78.9%)	124 (63.3%)	434 (83%)	402 (86.5%)	143 (85.6%)	177 (87.2%)	3.4 × 10^−5+++^
	Hyperglycemia /DM2	804 (59.6%)	134 (36.9%)	496 (68.6%)	2.2 × 10^−16+++^	517 (58.4%)	67 (34.2%)	343 (66%)	287 (61.7%)	67 (40.1%)	153 (75.4%)	0.01^+^
	Low-HDL	905 (67%)	275 (75.6%)	452 (62.5%)	9.1 × 10^−6+++^	601 (67.9%)	153 (78.1%)	329 (63.3%)	304 (65.4%)	122 (73%)	123 (60.6%)	0.27
	Hyper- triglyceridemia	522 (38.7%)	140 (38.6%)	264 (36.5%)	0.97	308 (34.8%)	62 (31.6%)	172 (33%)	214 (46%)	78 (46.7%)	92 (45.3%)	3.1 × 10^−5+++^

Cohort composition and comparison of metabolic characteristics (profile and components) in the entire cohort (Total), and in subsets of women and men patients. In the table, numbers are shown with the respective% of subjects in the entire cohort and in the 2 age groups (range 19–45 years and range 55–85 years). The same representation is shown in sexes subsets (women and men). By the same cohort stratification, the number and respective% of subjects MHO, MetS, and its subset MetS.5 and the number and respective% of subjects affected by each metabolic component are shown in all ages and in specific age ranges. The% of each MetS.5 subset was calculated in relation to the corresponding MetS group. Mantel–Haenszel tests were used to test the differences in % of subjects affected by metabolic components: between age ranges net of sex (*p*-value age ranges), between men and women adjusted for age ranges (*p*-value women vs. men). As an example: % of subjects who present hypertriglyceridemia does not change significantly between the 2 age ranges but it changes significantly with sex, indicating a strong influence of sex but not of age for this metabolic component (underlined values). Significance: *p* < 0.05 (+), 0.001 (+++). Abbreviations: = MHO, metabolically healthy obese status; MetS, metabolic syndrome status; MetS.5, subset of metabolic syndrome status which presents all 5 metabolic syndrome components; range 19–45 years, fertile age; range 55–85 years, postmenopausal status; W, Women; M, Men; vs., versus.

## Data Availability

The data presented in this study are available on request from the corresponding author. The data are not publicly available due to ethical reasons.

## Supplementary Materials

The following supporting information can be downloaded at: https://www.mdpi.com/article/10.3390/metabo12050419/s1 Figure S1: The representative spectrum 1H NMR obtained from serum samples of Piancavallo subjects; Table S1: Characteristics of Piancavallo cohort by anthropometric measures.
