# Supplementary material for: Sex Dimorphism in the Metabolome of Metabolic Syndrome in Morbidly Obese Individuals
Source: Metabolites. 2022 May 6;12(5):419. doi: 10.3390/metabo12050419 (PMC9147578; doi:10.3390/metabo12050419)
Supplement: Supplementary file 1 [file metabolites-12-00419-s001.zip › metabolites-1707081-supplementary.pdf]

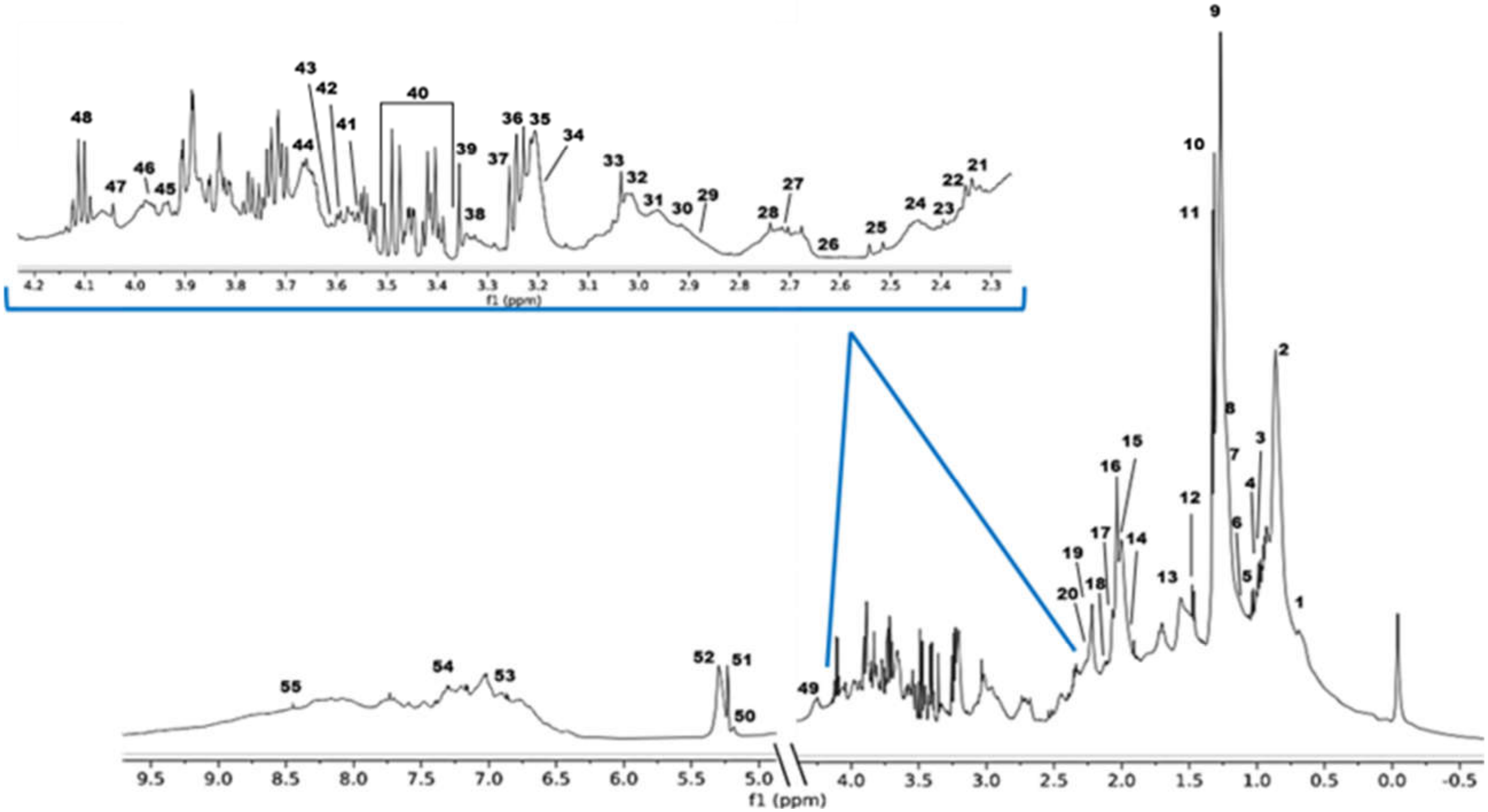

Figure S1. The representative spectrum 1H NMR obtained from serum samples of Piancavallo subjects. The following metabolites are identified: 1, Cholesterol; 2, FA1 ; 3, Leucine; 4, Isoleucine; 5, Valine; 6, 2-oxobutyrate; 7, Ethanol; 8, 3-hydroxybutyrate; 9, FA2; 10, VLDL2; 11, LDL2; 12, Alanine; 13, FACO1; 14, Acetate; 15, PUFAs; 16, NAC1; 17, NAC2; 18, Proline; 19, FACO2 ; 20, Acetone; 21, Glutamate; 22, Pyruvate; 23, Succinate; 24, Glutamine; 25, Citrate; 26, Methionine; 27, Dimethylamine; 28, Sarcosine; 29, Trimethylamine; 30, Dimethylglycine; 31, UFA1; 32, Lysine; 33, Creatine; 34, Choline; 35, CCC; 36, Arginine + Glucose; 37, TMAO; 38, 2-oxosuccinate; 39, Methanol; 40, Glucose; 41, Glycine + Glucose; 42, o-phosphocholine; 43, myo-inositol; 44, Methylhistidine; 45, Glycolate; 46, Serine; 47, Creatinine; 48, Lactate; 49, Threonine; 50, Mannose+Glycogen fragments; 51, Glucose; 52, UFA2; 53, Tyrosine; 54, Phenylalanine; 55, Formate. Key for NMR moieties: FA: fatty acids; FA1: CH3-; FA2: -CH2-; FACO1: -CH2CO; FACO2: -CH2CH2CO; UFA1: =CHCH2CH2-; UFA2 =CHCH2-; PUFAs: =CHCH2CH=; CCC: choline-containing compounds; TMAO: trimethylamine oxide; NAC1/2: acetyls in glycoproteins.

Anthropometric Measures (mean ± st.dev)		Total			Women			Men		
		MHO	MUHO	*p-value*	MHO	MUHO	*p-value*	MHO	MUHO	*p-value*
**Waist Circumference (cm)**	**all ages**	128,9 ± 15,3	137,1 ±18,9	*1,4E-15****	123,7 ± 11,4	132,1 ± 11,5	*7,6E-11****	144 ± 15,4	144,4 ± 13,8	*0,87*
	**range 19-45 y**	130,9 ± 18,1	143,8 ± 14,1	*4,1E-05****	122,9 ± 13,9	137,5 ± 13,5	*4,5E-04****	145 ± 16,1	146,8 ± 13,6	*0,63*
	**range 55-85 y**	127,3 ± 11,4	134,1 ± 12,3	*8,6E-06****	124,7 ± 10,2	131,1 ± 1,2	*2,7E-05****	139,9 ± 12,4	141 ± 13,1	*0,56*
**Waist to hip ratio**	**all ages**	0,92 ± 0,1	0,98 ± 0,1	*1,4E-15****	0,89 ± 0,1	0,94 ± 0,1	*9,4E-12****	1 ± 0,1	1,04 ± 0,1	*0,0022***
	**range 19-45 y**	0,92 ± 0,1	1,02 ± 0,1	*8,9E-08****	0,88 ± 0,1	0,92 ± 0	*0,0231**	1 ± 0,1	1,06 ± 0,1	*0,0015***
	**range 55-85 y**	0,92 ± 0,1	0,97 ± 0,1	*6,6E-08****	0,9 ± 0,1	0,94 ± 0,1	*8,7E-06****	1 ± 0,1	1,03 ± 0,1	*0,087*
**BMI (kg/m²)**	**all ages**	47 ± 6,2	48,1 ± 6,5	*0,036**	46,5 ± 5,6	48,4 ± 6,5	*0,0038***	48,4 ± 7,4	47,8 ± 6,5	*0,59*
	**range 19-45 y**	47,9 ± 7,3	50,4 ± 6,8	*0,054*	46,6 ± 6,5	51,7 ± 7,2	*0,010**	50,1 ± 8	49,7 ± 6,5	*0,83*
	**range 55-85 y**	45,6 ± 4,6	46,7 ± 5,9	*0,096*	46 ± 4,8	47,6 ± 5,7	*0,025**	44,8 ± 5,4	45,7 ± 6,3	*0,56*

Table S1. Comparison of anthropometric measures of MHO and MUHO subjects by age ranges in the Total and in subsets of and Men. *Mean values and standard deviation of Waist circumference, Waist to hip ratio and BMI were calculated for each group. t-tests were used to test the significance of the differences in subjects MHO and MUHO in different sexes and age ranges (19-45 y; 55-85 y). Waist circumference and Waist to hip ratio indicate a strong relationship between fat tissue and its distribution and MetS.5. Waist circumference and Waist to hip ratio values show the most significance in total population and in Women subgroup, regardless of age. Whereas only Waist to hip ratio shows statistical significance in Men group regardless of age. On the contrary, BMI lacks significance or appears only slightly related with MUHO in both Men and Women. Significance: (*) p < .05, (**) p < .01, (***) p < .001.*

Abbreviations: = MHO, metabolically healthy obese status; MUHO, metabolically unhealthy obese status; BMI, Body mass index; range 19-45 y, fertile age; range 55-85 y, postmenopausal status.
